# Activation of innate immune genes in caprine blood leukocytes after systemic endotoxin challenge

**DOI:** 10.1186/s12917-016-0870-x

**Published:** 2016-10-28

**Authors:** Øyvind Salvesen, Malin R. Reiten, Peter M. H. Heegaard, Michael A. Tranulis, Arild Espenes, Kerstin Skovgaard, Cecilie Ersdal

**Affiliations:** 1 Faculty of Veterinary Medicine and Biosciences, Norwegian University of Life Sciences, Oslo, Norway; 2 Innate Immunology Group, Section for Immunology and Vaccinology, National Veterinary Institute, Technical University of Denmark, Frederiksberg, Denmark

**Keywords:** Interferon stimulated genes, Extrahepatic acute phase proteins, Systemic inflammation, Lipopolysaccharide (LPS), Endotoxemia, Blood leukocyte gene expression, Innate immunity, Goat

## Abstract

**Background:**

Sepsis is a serious health problem associated with a range of infectious diseases in animals and humans. Early events of this syndrome can be mimicked by experimental administration of lipopolysaccharides (LPS). Compared with mice, small ruminants and humans are highly sensitive to LPS, making goats valuable in inflammatory models. We performed a longitudinal study in eight Norwegian dairy goats that received LPS (0.1 μg/kg, *Escherichia coli* O26:B6) intravenously. A control group of five goats received corresponding volumes of sterile saline. Clinical examinations were performed continuously, and blood samples were collected throughout the trial.

**Results:**

Characteristic signs of acute sepsis, such as sickness behavior, fever, and leukopenia were observed within 1 h of LPS administration. A high-throughput longitudinal gene expression analysis of circulating leukocytes was performed, and genes associated with the acute phase response, type I interferon signaling, LPS cascade and apoptosis, in addition to cytokines and chemokines were targeted. Pro-inflammatory genes, such as IL1B, CCL3 and IL8, were significantly up-regulated. Interestingly, increased mRNA levels of seven interferon stimulated genes (ISGs) were observed peaking at 2 h, corroborating the increasing evidence that ISGs respond immediately to bacterial endotoxins. A slower response was manifested by four extrahepatic acute phase proteins (APP) (SAA3, HP, LF and LCN2) reaching maximum levels at 5 h.

**Conclusions:**

We report an immediate induction of ISGs in leukocytes in response to LPS supporting a link between the interferon system and defense against bacterial infections. The extrahepatic expression of APPs suggests that leukocytes contribute to synthesis of these proteins at the beginning of a systemic inflammation. Taken together, these findings provide insights into the dynamic regulation of innate immune genes, as well as raising new questions regarding the importance of ISGs and extrahepatic APPs in leukocytes after systemic endotoxin challenge.

**Electronic supplementary material:**

The online version of this article (doi:10.1186/s12917-016-0870-x) contains supplementary material, which is available to authorized users.

## Background

Sepsis is a life-threatening condition caused by a dysregulated host response that can occur in a range of bacterial, viral, or toxic diseases in animals and humans [1]. Initial events of this syndrome can be mimicked by experimental administration of LPS, derived from the cell-wall of gram-negative bacteria. The host response to LPS is complex and reveals major species differences [2–4]. Interestingly, some species such as mice and the Rhesus monkey, have developed tolerance by limiting harmful inflammation even when the pathogen loads are high [4]. At the other end of the sensitivity spectrum are humans, rabbits and small ruminants, which are extremely sensitive to endotoxins [2–4]. This marked difference in sensitivity towards LPS has led scientists to question the validity of extrapolation of rodent inflammatory responses to human medicine [5].

LPS is a potent stimulator of the innate immune system that provides the first line of defense against pathogens and initiates the acute phase response (APR) [6]. Among leukocytes, circulating monocytes and neutrophils are the primary target cells involved in protection against LPS. These cells constitutively express membrane-bound CD14 and Toll-like receptor 4 (TLR4), important for endotoxin recognition and activation of the innate immune system [6, 7]. Upon LPS stimulation, the CD14-MD-2-TLR4-complex initiates signaling through MyD88-dependent or TRIF-dependent pathways [8], leading to the expression of pro-inflammatory cytokines, chemokines and enzymes [6, 9, 10]. The ensuing physiological response culminate in a behavioral state referred to as sickness behavior [11].

Activated immune cells release pro-inflammatory mediators, such as IL-1, IL-6, IL-8 and TNF-α, which stimulate the liver to produce positive acute phase proteins (APPs) [12] and simultaneously down-regulate negative APPs [13]. The major APPs in goat, serum amyloid A (SAA) and haptoglobin (HP), serve several functions in the APR [14] and are valuable diagnostic indicators of inflammation [13, 15]. APPs are also released from extrahepatic tissues such as mammary gland [16, 17], lungs [18], adipose tissue [19, 20], colon [20], and lymphoid organs [12], but their patho-physiological significance is incompletely understood. The role of APPs released from leukocytes during infections or endotoxemia has been the subject of only a few previous studies [12].

Increasing evidence show that LPS induces transcription of interferon stimulated genes (ISGs), originally considered exclusively anti-viral, but with accumulating data indicating a range of other immunomodulatory properties [21]. The in vivo regulation of these genes in leukocytes, however, has not been described in detail.

In 2013, the worldwide population of small ruminants comprised about 1006 million goats and 1073 million sheep [22]. Large herds and intensive production increase the number of endotoxin-related diseases such as acute ruminal acidosis, per-acute mastitis, toxic metritis, and septic peritonitis. Additionally, failure of, or insufficient, passive transfer of colostral immunoglobulins is a common cause of neonatal sepsis in lambs and goat kids [23]. Circulating leukocytes play a crucial role in initiating the APR in all sepsis-related diseases. Thus, investigation of changes in blood leukocyte gene expression will provide a better understanding of the biological processes during endotoxemia. In a microarray study of human mononuclear cells, more than 800 genes were differentially expressed 6 h post challenge, highlighting the complexity of the response to systemic endotoxins [24]. Here, we report a longitudinal in vivo LPS study in goat comprising clinical, biochemical, and hematological responses, as well as leukocyte transcriptional profiles of selected immune genes.

## Methods

### Goats

A total of 13 Norwegian dairy goats, non-pregnant females, were recruited from a research herd at the Norwegian University of Life Sciences (NMBU). The mean age ± SD was 7.1 ± 1.8 months and the mean body weight ± SD was 25.1 ± 4.1 kg. Before the experiment, the goats were housed for at least 21 days to acclimatize. They were kept under a 16 h light/8 h dark cycle and housed in groups of 2–4 goats. Hay and water was provided *ad libitum*, and they were fed a commercial goat pellet concentrate twice a day. During this period, a full clinical examination was performed three times, and all goats were clinically normal. Fecal parasite egg counts were low and hematology was within reference values before the experiment.

### LPS challenge

LPS (*Escherichia coli* O26:B6, L2654 Sigma-Aldrich, USA) was diluted in 0.9 % sterile saline to a concentration of 1.5 μg/ml. The goats were divided into two groups as follows: Eight goats receiving 0.1 μg/kg LPS intravenously, and a control group of five goats receiving corresponding volumes of sterile saline. The dosage was based on existing literature [25–27] and a pilot titration study involving three animals (data not included).

### Clinical examination

After LPS challenge, clinical examination was performed at 12 time points during the first 7 h and once the next morning (24 h). The general condition was determined evaluating body posture (standing, lying), head- and ear-position, pupil size, appetite, grooming, shivering and social interaction. Respiratory frequency was recorded by observation, and ruminal contraction and heart frequency by auscultation. To ensure accurate rectal temperatures, all measurements were repeated three times at each time point. Clinical examination was performed at corresponding time points in control animals.

### Blood samples

Blood samples (EDTA, whole blood and PAX blood tubes) were drawn from *v. jugularis* using a vacutainer system (BD Company, USA). Baseline samples (0 h) were taken within half an hour of the LPS challenge. The other sampling times were 1 h, 2 h, 5 h and 24 h after LPS administration. To investigate if handling stress itself affects the quantified parameters, two blood samples were taken from the controls, a baseline sample (0 h) before saline administration and another sample 1 h later.

### Hematological and biochemical analysis

A complete blood cell count including differential count was performed immediately after sampling using the ADVIA 120 Hematology system (caprine analyzing program) (Siemens, Germany). Whole blood was centrifuged and serum stored at −20 °C until biochemical analysis. Serum total protein, albumin and glucose were analyzed by ABX Pentra 400 (Horiba, France). Circulating levels of serum amyloid A (SAA) were analyzed by an ELISA method (Tridelta multispecies assay kit, Ireland) at three of the sampling time points (0 h, 5 h and 24 h).

### RNA isolation

After blood sampling, PAX-gene blood RNA tubes (PreAnalytiX, Switzerland) were gently inverted 8–10 times. The tubes were incubated overnight at room temperature followed by storage at −80 °C. The isolation of total RNA was performed according to the manufacturer’s instructions using PAXgene Blood miRNA kit (Qiagen, Germany). Isolated RNA was quantified at optical density (OD) 260_,_ and purity evaluated by OD260/280 and OD260/230 ratios using DeNovix DS-11 spectrophotometer. RNA integrity was analyzed by RNA 600 Nano chips in compliance with the Bioanalyzer 2100 system (Agilent, USA) and each sample was assigned a RNA integrity number (RIN) from 1 to 10, with 10 being non-degraded RNA. The mean RIN value of included samples ± SD was 9.1 ± 0.30. All samples were treated with DNase while bound to columns to remove any contaminating genomic DNA, and stored at −80 °C.

### cDNA synthesis

Two separate cDNA replicates were made for each sample. 250 ng of total RNA was converted into first strand cDNA using QuantiTect Reverse Transcription Kit (Qiagen, Germany) according to the manufacturer’s instructions. A non-reverse transcriptase control (NoRT) and no template control (NTC) were included, and all cDNA samples were stored at −20 °C.

### Primer design

A total of 44 genes associated with the LPS signaling cascade, early pro-inflammatory response, cytokines, chemokines, ISGs, APPs and apoptosis were chosen for investigation. Primer sequences and gene abbreviations can be found in Additional file 1.

Nucleic acid sequences were obtained from online genome databases and primers were designed by the Primer 3 software [28]. For previously untested primer assays, two primer pairs were designed for each transcript to validate that the correct transcript was being amplified. Additionally, the primer specificity was verified *in silico* using nucleic BLAST search against the *Capra hircus* genome. Primers were synthesized by Sigma-Aldrich (Germany). When possible, primers were designed to span exon/exon boundaries and to cover all known splice variants.

### Fluidigm biomark HD qPCR

#### Preamplification and endonuclease treatment

A preamplification of target genes was performed to ensure adequate amounts of templates for high-throughput quantitative real time PCR (qPCR). Equal amounts of all primers used in the study were pooled in a 200 nM primer mix in low Tris-EDTA (TE) buffer, pH 8.0. A total of 10 μl comprising 2.5 μl primer mix, 2.5 μl of cDNA and 5 μl of TaqMan PreAmp was prepared per sample. Preamplification was carried out in a thermal cycler using the following program: Initial denaturation for 10 min at 95 °C followed by 20 cycles of 15 s at 95 °C and 4 min at 60 °C for annealing and elongation. To prevent carry-over of unincorporated primers after preamplification, 4 μl of 4U/μl exonuclease was added to the samples and incubated for 30 min at 37 °C and 15 min at 80 °C. Finally, cDNA was diluted 1:5 in TE buffer before qPCR.

#### Preparation of primer and sample assays

For each primer assay, a primer mix consisting of 3 μl of 2X Assay loading Reagent (Fluidigm, USA) and 3 μl of 20 μM specific forward/reverse primer was prepared. A sample mix consisting of 3 μl 2X TaqMan Gene Expression Mastermix, 0.3 μl 20X DNA binding Dye, 0.3 μl EvaGreen 20X, 0.9 μl TE buffer and 1.5 μl preamplified and exonuclease treated cDNA was made for each sample line.

#### Dynamic array qPCR analysis

Preparation and loading of Fluidigm 96.96 Dynamic Array IFC (integrated fluidic circuit), which combines 96 samples with 96 primer assays in 9216 simultaneous reactions, was performed according to manufacturer’s instructions and as previously described [29]. Using Fluidigm Real-Time Analysis software 3.0.2, expression data were visualized as a heat map based on Cq values. All amplifications and melting curves were evaluated and only genes with a single melting peak were accepted. Each chip included a NTC, a NoRT and three interplate calibrators. NTCs and melting curves were used to assess non-specific amplification or sample contamination. NoRT controls were used to evaluate potential genomic DNA background signals. No sample contamination or interfering genomic DNA signals were detected.

For each primer assay, a pool of all preamplified and exonuclease-treated cDNA samples were used to make three separate dilution series with the following dilutions: 1:2, 1:10, 1:50, 1:250 and 1:1250. Standard curves were constructed to obtain primer amplification efficiencies, correlations and dynamic range. To cover the dynamic range of genes with low expression, an additional standard dilution was made using four “high responding” samples. In 6 of the 96 primer assays, construction of standard curves was not possible and they were not included in further analyses. Primer efficiencies of included assays varied between 0.95 and 1.11 and had a correlation coefficient above 0.95. Four of the genes (IL6, IL12, CCL20 and MMP8) and one reference gene (ACACA) had very low expression in the samples and not subject to further investigation.

### Light cycler 480 qPCR

The expression of IFNB1 gene was investigated by Light cycler 480 qPCR using SYBR Green PCR Master Mix under the following conditions: Initial denaturation for 5 min at 95 °C, followed by 40 amplification cycles (10 s at 95 °C, 10 s at 60 °C and 15 s at 72 °C) and construction of melting curves. IFNB1 primer sequences were adapted from [30] and can be found in Additional file 1.

### Preprocessing of data

Heat map data were analyzed using GenEx5 software (MultiD, Sweden). First, interplate calibration was performed and each primer assay was corrected for primer efficiencies. Six potential reference genes (HPRT 1, HMBS, ACTB, HSP90AA1, ALAS 1 and GADPH) were validated using the integrated geNorm and Normfinder software in GenEx5. HPRT1, HMBS, ACTB and HSP90AA1 were used for the final normalization. Three of the samples were excluded due to high variation between cDNA replicates. After averaging cDNA duplicates, genes with more than 15 % missing data (IL10, INFG and CASP3) were removed and the remaining missing data (1.6 %) were replaced with the highest Cq value +1. Expression levels for each gene were then scaled to 1 for the sample with lowest expression, and all other samples for that specific gene calculated relative to this. Finally, expression data were transformed from Cq (log2) to relative quantities (relative fold change, linear scale).

### Descriptive and statistical analysis

Data are presented as mean ± standard error of the mean (SEM). Graphical and statistical analyses were performed in GraphPad Prism 6 (GraphPad software Inc., USA) and Microsoft Excel 2013. To account for multiple comparisons, a one-way ANOVA and Dunnett’s post hoc test was performed on Log2 transformed expression data. Differential expression was assessed by a limit of ± 2 fold change in expression compared with baseline samples and *p* values with the following significance levels: *P* < 0.05; *P* < 0.01; *P* < 0.001.

## Results

### Clinical and hematological responses

**Fig. 1:**
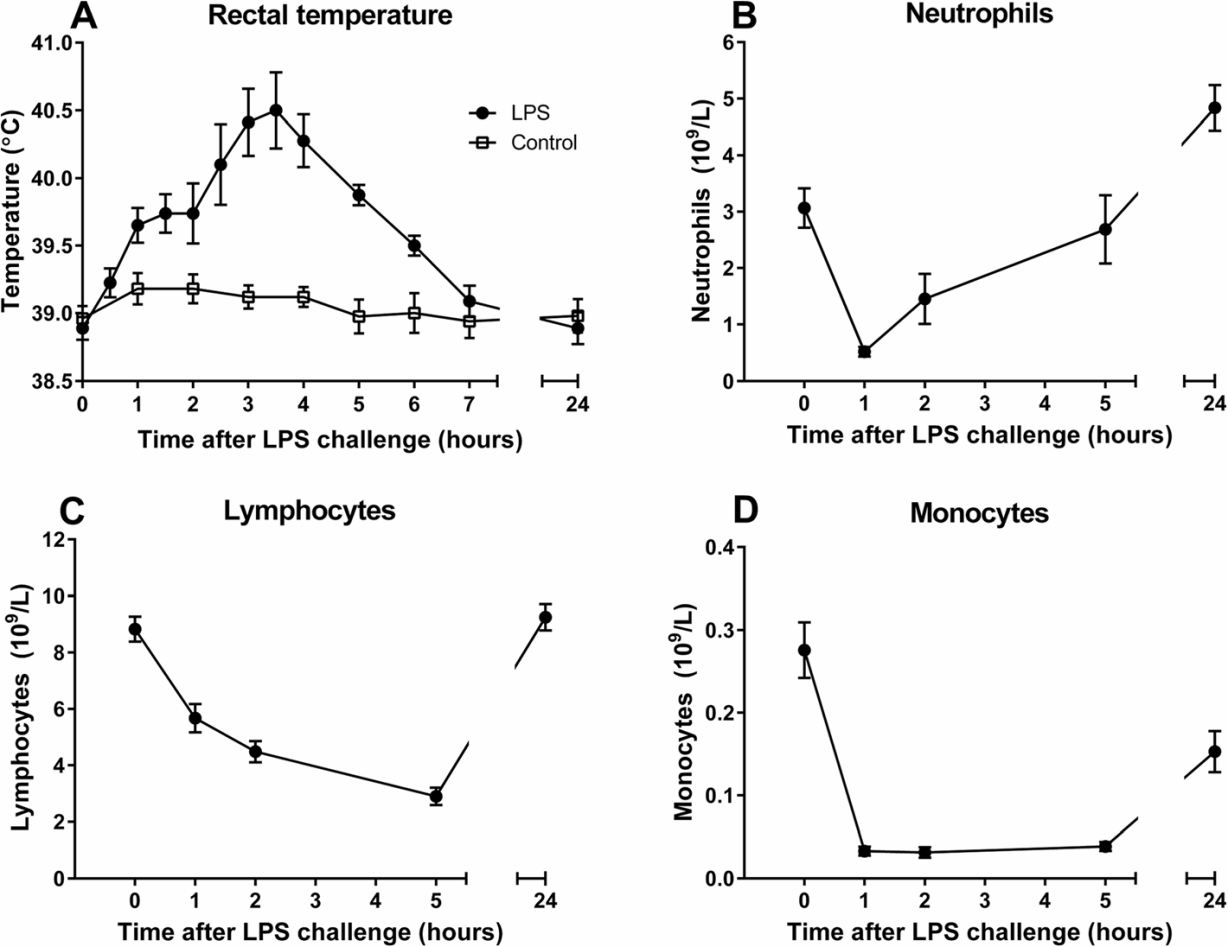
Rectal temperature and hematology after systemic LPS challenge. Rectal temperature (**a**), and blood leukocyte count including neutrophils (**b**), lymphocytes (**c**) and monocytes (**d**) following 0.1 μg/kg intravenous LPS administration. Values are mean ± SEM, *n* = 8

The mean rectal temperature increased in a biphasic manner from 38.9 °C (±0.09) to 40.5 °C (±0.28) 3.5 h after LPS injection (Fig. 1). Within the first 45 min, the goats displayed tachypnea, head shaking, anorexia and reduced locomotor activity, accompanied by a period of shivering, lasting 15–25 min. In five of the goats, a second period of shivering was observed between 1 h 45 m and 2 h 30 m after LPS administration. Also, all goats displayed an elevated heart frequency and decreased rumen motility. Overall, clinical signs were most prominent during the first five hours, which correlated with profound leukopenia, acute in neutrophils and more gradual in lymphocytes. The number of basophils and eosinophils declined significantly (data not shown), and a distinct monocytopenia was observed throughout all post-injection time points (Fig. 1). The goats gradually improved and by 7 h post injection all were considered clinically normal, but with an elevated heart frequency. The number of neutrophils had normalized at 5 h post challenge, but was elevated at 24 h compared with baseline levels. None of the control animals displayed alterations in clinical or hematological parameters during the experimental period.

### Blood chemistry

**Fig. 2:**
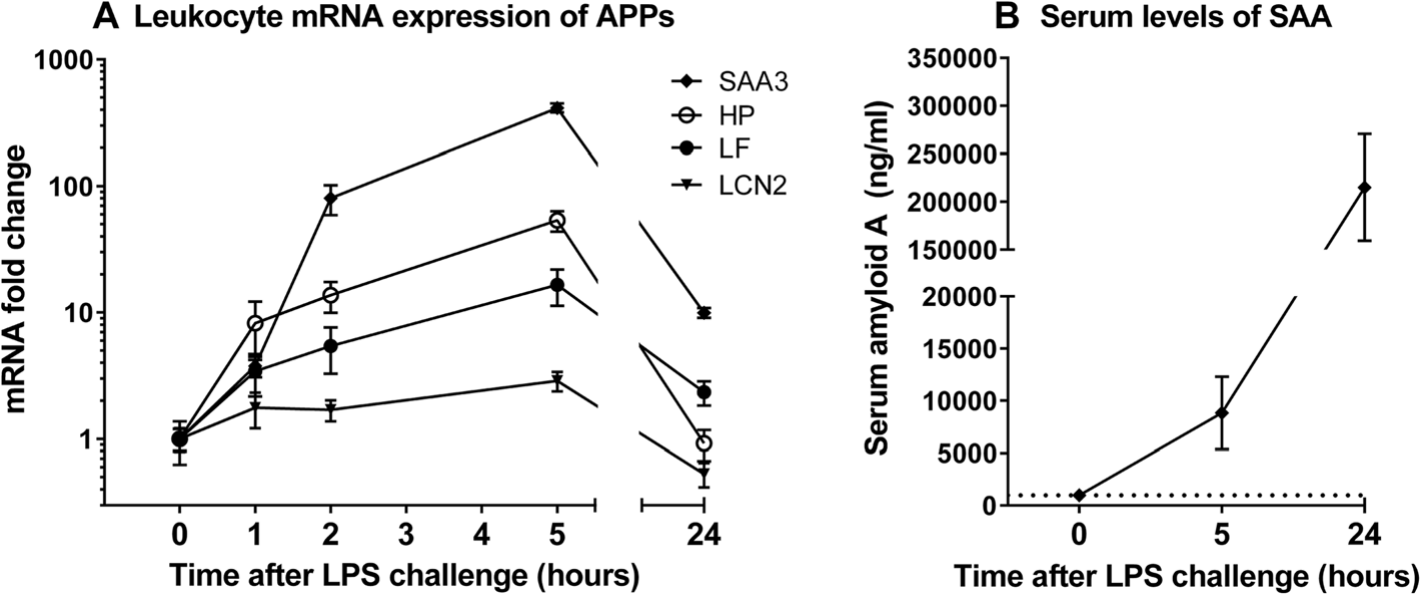
Acute phase protein (APP) genes and serum levels of SAA after systemic LPS challenge. **a** Relative mRNA expression of serum amyloid A3 (SAA3), haptoglobin (HP), lactoferrin (LF) and lipocalin 2 (LCN2), compared with baseline levels (0 h) scaled to 1. Real time qPCR was performed on RNA extracted from circulating leukocytes at the indicated time points. Results are shown as mean relative fold change ± SEM. **b** Circulating levels of serum amyloid A (SAA) shown as mean serum concentration (ng/ml) ± SEM. The y-axis dotted line indicates the lower detection limit of the ELISA (470 ng/ml). *n* = 8

Total protein and albumin decreased throughout the experiment, and albumin levels were significantly reduced 24 h after LPS injection. Circulating SAA was below detection limit (470 ng/ml) in all animals before challenge, and reached 215 000 ± 55 600 ng/ml at 24 h (Fig. 2). Serum glucose increased significantly towards 2 h, followed by a decrease at 5 h, but all values were within the reference range.

### Gene expression analysis

The regulated genes and fold changes are summarized in Table 1. The magnitude of the fold change reflects the gene expression alterations relative to baseline levels (0 h) scaled to 1. Of the 37 target genes analyzed after preprocessing, 28 were significantly regulated at least at one time point after LPS injection, with a minimum fold change of ± 2 compared with baseline levels. Expression levels of TNF, BPI, BCL2, BAX, ITGAM, ANAX1, C3 and STIP1 were not significantly altered at any of the time points.

**Table 1:** Fold change in gene expression of selected immune genes after LPS challenge

	Gene	mRNA fold change				*P*		Gene	mRNA fold change				*P*
		1 h	2 h	5 h	24 h				1 h	2 h	5 h	24 h	
Interferon stimulated genes	CXCL10	4	**85**	11	2	**<0.001**	LPS-pathway	TLR4	−2	2	**3**	1	**<0.05**
	ISG15	2	**43**	17	4	**<0.001**		CD14	**−4**	1	1	1	**<0.01**
	ISG20	1	**9**	7	3	**<0.001**		MyD88	**−3**	1	1	1	**<0.05**
	IFIT1	1	**9**	3	1	**<0.01**		TRAF6	1	−2	−**2**	−2	**<0.01**
	OAS1	1	**14**	7	2	**<0.001**	IFN-pathway	STAT1	1	**2**	2	1	**<0.001**
	IFI6	1	5	**5**	3	**<0.001**	Cytokines and chemokines	IL1B	2	2	**3**	2	**<0.01**
	MX1	1	**17**	11	2	**<0.001**		IL8	2	**3**	2	1	**<0.01**
Acute phase proteins	SAA3	4	80	**412**	10	**<0.001**		CCL3	3	2	**4**	1	**<0.001**
	LF	3	5	**17**	2	**<0.001**		CCL5	−4	−3	−1	**−5**	**<0.001**
	HP	8	14	**54**	1	**<0.001**		IL18	−3	**−3**	−3	1	**<0.01**
	LCN2	2	2	**3**	−2	**<0.01**		IL1RN	1	**14**	7	1	**<0.05**
Enzymes	HSPA1A	1	3	**13**	1	**<0.001**	Other immune- related genes	TICAM	1	−3	**−3**	−2	**<0.001**
	S100A9	1	8	**18**	3	**<0.001**		ITGB2	−2	−2	−**4**	1	**<0.001**
	SOD2	−3	3	**3**	1	**<0.001**		MHCII	1	−2	**−3**	1	**<0.001**

Relative gene expression following intravenous LPS (0.1 μg/kg) administration in goats. Fluidigm qPCR was performed on RNA extracted from circulating leukocytes at the indicated time points. Results are expressed as mean fold change relative to baseline samples (0 h) scaled to 1. The most regulated time point for each gene is highlighted in bold, and significance level at this time point is given

**Fig. 3:**
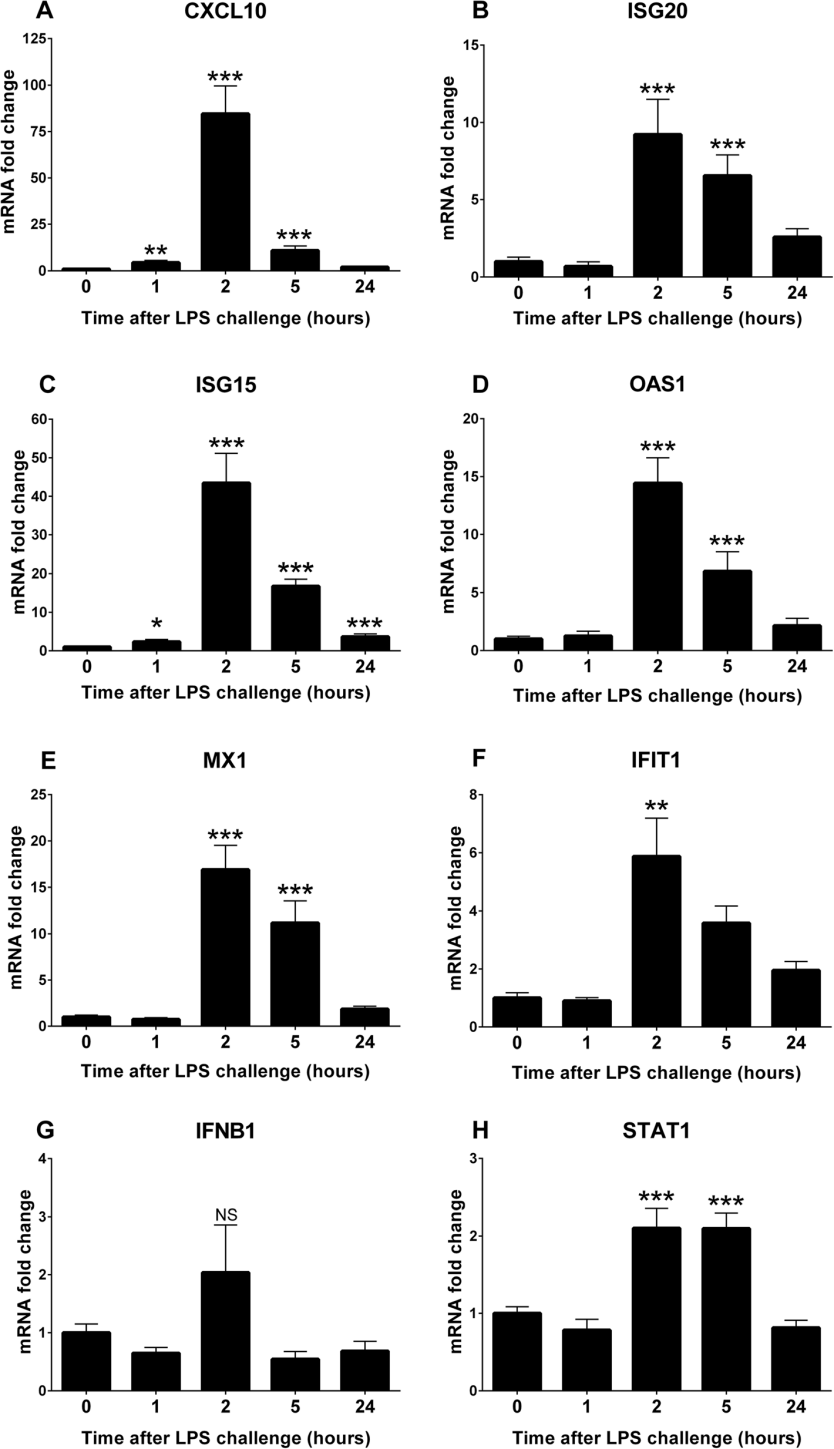
Gene expression of leukocyte ISGs (**a**-**f**), IFNB1 (**g**) and STAT1 (**h**) after systemic LPS challenge. Relative mRNA fold change compared with baseline levels (0 h) scaled to 1. Real time qPCR was performed on RNA extracted from circulating leukocytes at the indicated time points. Results are expressed as mean relative fold change ± SEM, *n* = 8. NS = not significant. *P < *0.05*; **P < *0.01*; ***P < *0.001*

The transcription of CXCL10 and ISG15 increased already 1 h after LPS challenge. All ISGs (CXCL10, ISG15, IFI6, ISG20, OAS1, IFIT1 and MX1) and STAT1, involved in interferon signaling [31], were significantly up-regulated at 2 h and 5 h post injection. IFNB1 increased of about 2-fold at 2 h, but this was not statistically significant. The expression of IFI6 and ISG15 remained elevated at 24 h, but only with a fold change of 3 and 4, compared with baseline levels (Fig. 3).

The mRNA levels of APP genes, HP, LF, LCN2 and SAA3 increased 1 h post injection, and peaked at 5 h before returning to baseline levels at 24 h, except SAA3 which remained up-regulated (Fig. 2). SAA3 was by far the most up-regulated gene, reaching a fold change of 412 at 5 h post LPS challenge, and its expression strongly correlated with that of S100A9 (*R* = 0.90), which is involved in leukocyte extravasation [32]. Expression levels of LPS pathway genes (TLR4, CD14, MyD88, TRAF6) were stable throughout the experiment, however TLR4 was slightly (3-fold) up-regulated after 5 h. Three early pro-inflammatory cytokines (IL1B, IL8 and CCL3) were up-regulated during the first few hours.

Overall, genes involved in the LPS pathway, cell migration, and apoptosis displayed fold changes below 5, whereas most ISGs, APPs and genes involved in the later response were regulated by more than 10-fold.

## Discussion

Despite the many endotoxin-related diseases in small ruminants and the advantage as a model for human endotoxemia, studies of LPS-induced inflammation are limited in these species. In the present study we describe 28 genes that were significantly (*±2 fold change*, *p* < 0.05) regulated in blood leukocytes during a 24 h experimental period.

Interestingly, all ISGs investigated were significantly up-regulated, peaking already at 2 h after LPS injection. Of these, CXCL10 showed the greatest up-regulation with an 85-fold increase compared with baseline levels. In murine peripheral leukocytes, a 2-fold to 3-fold increase in the expression of CXCL10 and IFIT1 was observed 48 h after a single intraperitoneal LPS administration, but the immediate response was not studied [33].

ISGs, which are induced by type I interferon (IFN) activation, are multifunctional genes traditionally ascribed important roles in anti-viral defense, including chemotaxis, cell differentiation, and apoptosis [21, 34, 35]. The fact that bacterial products such as LPS stimulate the expression of ISGs, has led to experiments addressing the role of type I IFN signaling during bacterial challenge. Two different lines of transgenic mice with genetic knock-out of IFN-β or the IFN-α/β receptor, displayed reduced survival against both streptococci and *Escherichia coli* compared with wild type mice [36]. This suggests an essential role of type I IFN-signaling in host resistance against both gram-positive and gram-negative bacteria. Previous in vitro studies describe a TLR4-mediated IFNβ-dependent induction of ISGs in monocyte-derived macrophages [37–39] and an IFN-independent induction of ISGs (MX1 and ISG15) in neutrophils [38]. However, transcriptional activity in neutrophils is considered low compared with other leukocytes [40], and their contribution to the total ISG pool is presumably limited. Although STAT1 phosphorylation levels were not analyzed in this study, we observed increased expression of STAT1, suggesting that interferon signaling was stimulated. IFNB1 was not significantly altered, but as leukocytes expressing IFNB1 and STAT1 extravasate after LPS challenge, the initial transcription levels of these genes are probably underestimated. Thus, a subtle or transient induction of IFNB1 will be difficult to detect in our model. To our knowledge, the present study is the first report of an immediate induction of ISGs in leukocytes upon LPS challenge, and it further supports a link between interferon signaling and defense against bacterial infections [21, 36, 39].

APPs are a group of proteins that undergo substantial quantitative changes following external or internal stress, such as inflammation, neoplasia, or trauma [13]. As part of the innate immune system, these proteins contribute in basic defense mechanisms. Although APPs are mainly generated by the liver, increasing evidence indicate a local production in a number of tissues and cell types [12, 16–20, 41, 42]. Strikingly, mRNA levels of serum amyloid A3 (SAA3), lactoferrin (LF), haptoglobin (HP) and lipocalin (LCN2) were up-regulated in leukocytes upon LPS stimulation in the present study. SAA3 had the highest expression, reaching more than 400 times the baseline level at 5 h post challenge. This molecule is one of several SAA isoforms and is thought to be involved in cholesterol metabolism, modulating the innate immune response, as well as being a monocyte chemoattractant [43]. Recombinant SAA3 is also reported to have antimicrobial activity against *Escherichia coli*, *Streptococccus uberis* and *Pseudomonas aeruginosa* in the bovine mammary gland [44]. In murine colon, SAA3 synthesis has been linked to the TLR4 signaling axis and expression of the SAA3 gene was significantly reduced in mice with genetic knockout of MyD88 [20]. HP, LF and LCN2, also have bacteriostatic properties [45–47], mediated by binding and sequestering of iron. The first two are synthesized during differentiation of neutrophilic granulocytes and stored in specific granules that can be released upon activation [42, 48]. LCN2 has recently been reported to be a major APP in rat and mouse, as mRNA levels dramatically increase in both leukocytes and liver following inflammation [46, 49, 50]. In the current study, LCN2 mRNA was modestly up-regulated (3-fold), suggesting that this gene should be considered a minor APP in caprine leukocytes.

It is not clear if the increased mRNA levels of SAA3, HP, LF and LCN2 in leukocytes contribute to elevated protein levels in serum, or if the proteins are secreted after extravasation of the leukocytes. It has been shown that HP can be released by neutrophils present in milk following intramammary administration of endotoxin [41]. Thus, it is plausible that activated leukocytes release APPs after extravasation in response to local stimulation. Baseline serum concentrations of SAA were below detection limit (470 ng/ml) in all eight goats and increased dramatically towards 24 h after LPS challenge, reaching a mean concentration of 215 000 ng/ml. Although the liver must be considered the main source of SAA, particularly SAA1 and SAA2, it cannot be excluded that leukocyte SAA3 contribute to the circulating pool since the measurements do not differ between the different isoforms of SAA. In a murine obesity model, increased SAA3 mRNA expression in adipocytes did not affect circulating SAA levels [51], but these cells are extravascular and cannot be directly compared to cells in blood. Whether stimulated blood leukocytes release their APP products while in circulation, or just prepare for secretion to occur once migrated into tissues, remains to be investigated.

The decreased expression of CD14 and MyD88 mRNA detected 1 h post injection, was unexpected because these genes are crucial for recognition of LPS and initiating TLR4 signaling [6]. In the present study, mRNA was extracted from total leukocytes, hence the composition of circulating white blood cells affects the mRNA levels. CD14 and MyD88 genes are profoundly expressed in neutrophils and monocytes, two cell types that were dramatically decreased at 1 h due to tissue extravasation (Fig. 1). Thus, the decreased mRNA levels probably reflect the reduced numbers of neutrophils and monocytes in the circulating cell population, rather than down-regulation due to LPS.

Among the early pro-inflammatory cytokines we report an increased expression of IL8, CCL3, and IL1B within the first hours after LPS administration, similar to that described in an equine model [52]. IL-8 and CCL3 primarily stimulate chemotaxis of granulocytes, as well as inducing phagocytosis at the site of infection, whereas IL1β is a key mediator of the inflammatory response, being involved in cell differentiation, proliferation and apoptosis [53]. Intravenous injection of IL1β induces sickness behavior and has been directly linked to fever [54] and anorexia [55]. Consequently, the increased levels of mRNA coding for IL1β is in agreement with the development of sickness behavior and fever manifested in the study. We also investigated the anti-inflammatory IL1 antagonist (IL-1RN) that inhibits IL1 activity by binding the IL1 receptors without generating signal transduction. Indeed, increased mRNA levels of IL-1RN were observed simultaneously with increased IL1B expression, exemplifying the tightly regulated innate immune response.

The clinical picture, with behavioral changes and alteration in parameters like temperature, heart rate, respiration and rumination described in the present study, corresponds with the duration of the reported changes in gene expression, and with previous clinical studies in goats [25–27]. These signs are characteristic of the acute phase of a systemic inflammatory response, and reflect the reorganization of the organism’s priorities to cope with infection. Notably, the behavioral changes demonstrate the profound effects systemic inflammation can have on the CNS [11].

## Conclusion

Our results demonstrate a brief and tightly regulated transcriptional response to systemic LPS administration in caprine leukocytes. Characteristic clinical signs of sepsis were accompanied by leukopenia and the induction of a range of immune-related genes. The increased mRNA levels of several ISGs substantiate the growing evidence that these genes possess multifunctional roles in the innate immune response. Extrahepatic expression of four APPs was also observed in caprine leukocytes, and increased dramatically upon stimulation, suggesting that leukocytes contribute to the synthesis of these proteins.

## Additional file

Additional file 1: Gene name, gene abbreviations and primer sequences used in the real time qPCR analysis. For previously untested primer assays, two primer pairs were designed for each transcript. (DOCX 22 kb)

## Abbreviations

APP: Acute phase protein
APR: Acute phase response
CNS: Central nervous system
ELISA: Enzyme-linked immunosorbent assay
ISG: Interferon stimulated gene
LPS: Lipopolysaccharide
NoRT: No reverse transcriptase
NTC: No template control
qPCR: Quantitative real-time polymerase chain reaction
RIN: RNA integrity number
