# Supplementary material for: Activation of innate immune genes in caprine blood leukocytes after systemic endotoxin challenge
Source: BMC Vet Res. 2016 Oct 28;12:241. doi: 10.1186/s12917-016-0870-x (PMC5084394; doi:10.1186/s12917-016-0870-x)
Supplement: Additional file 1: — Gene name, gene abbreviations and primer sequences used in the real time qPCR analysis. For previously untested primer assays, two primer pairs were designed for each transcript. (DOCX 22 kb) [file 12917_2016_870_MOESM1_ESM.docx]

| **Gene group** | **Gene name** | **Abbreviation** | **Primer sequence 5’ to 3’ (assay 1)** | **Primer sequence 5’ to 3’ (assay 2)** |
| --- | --- | --- | --- | --- |
| **LPS signaling pathway** | Toll like receptor 4 | TLR4 | F: TACCTGGAGTGGGAGGACAG  R: TGCATCTGCTGTTCCTTCTG | F: TCCAGGAAGGTTTCCACAAG  R: AGATACACCATCGGCTCTGG |
|  | Cluster of differentiation 14 | CD14 | F: CTCAGCGTGCTTGATCTCAG  R: GAAAGGGATTTCCGTTCACA | F: CCACCCTCAGTCTCCGTAAC  R: GTGTGCTTGGGCAATGTTC |
|  | Bactericidal permeability-increasing protein | BPI | F: CCCTCTTCCTGCTTGAGATG  R: CCAATCAGTCTGTCGGACCT | F: ATGGTGTACCTGGGCATCTC  R: CATGTCGTCTCTGAGGGTCA |
|  | Myeloid differentiation primary response 88 | MyD88 | F: TAAATGACATGGCAGGCATC  R: TCACATTCCTTGCTTTGCAG | F: GGATGGTGGTGGTTGTCTCT  R: ACCTGGAGAGAGGCTGAGTG |
|  | TNF receptor associated factor 6 | TRAF6 | F: AGATTGGCAACTTTGGGATG  R: CCGGGTTTGCCAGTATAGAA | F: TGCACAAGATGGAACTGAGG  R: TGGCATTGTGGACAACTCAT |
| **Cytokines/ Chemokines** | Tumor necrosis factor alpha | TNF | F: CCCTGGTACGAACCCATCTA  R: TATTCCGGCTGGTTGATCTC | F: CTCTTCTGCCTGCTGCACTT  R: GAACCAGAGGCCTGTTGAAG |
|  | Interleukin 1 beta | IL1B | F: CAGCCGTGCAGTCAGTAAAA  R: AGAGGAGGTGGAGAGCCTTC | F: AAAAGGTGATACGCCGACAC  R: GAAGACGAATCGCTTTTCCA |
|  | Interleukin 6 | IL6 | F: TGGAGGAAAAAGATGGATGC  R: CCAGAAGACCAGCAGTGGTT | F: TGAAGGAAAAGATCGCAGGT  R: ACCCACTCGTTTGAGGACTG |
|  | Interleukin 8 (CXCL8) | IL8 | F: ACCAACGGAAAAGAGGTGTG  R: TCTCTTCAAAAATGCCTGCAC | F: CCACACTGCGAAAATTCAGA  R: TCCTTGGGGTCTAAGCACAC |
|  | Interleukin 10 | IL10 | F: TGTTGACCCAGTCTCTGCTG  R: GCATCACCTCCTCCAGGTAA | F: GACATCAAGGAGCACGTGAA  R: AGGGCAGAAAACGATGACAG |
|  | Interleukin 12 | IL12 | F: ATTGAGGTCGTGATGGAAGC  R: TGGTTTGATGATGTCCCTGA | F: CAGGTCCAGGGAAAGAACAA  R: CTTGTGGCATGTGACTTTGG |
|  | Interleukin 18 | IL18 | F: TGGCAAGCTTGAACCTAAGC  R: CAGGTTGATTTCCCTGGCTA | F: AGCCAGGGAAATCAACCTGT  R: TGGTCTGGGGTGCATTATCT |
|  | Interferon gamma | IFNG | F: GATTCAAATTCCGGTGGATG  R: GATTTTGGCGACAGGTCATT | F: GGTCATTCAAAGGAGCATGG  R: CTCAGAGCTGCCGTTCAAG |
|  | Chemokine (C-C motif) ligand 3  Macrophage inflammatory protein 1α | CCL3 | F: TGGTGTCATCTTCCAGACCA  R: AGGTCGGTGACGTATTCCTG | F: CAGGAATACGTCACCGACCT  R: GAGGTCACTGGGTTCCTCAA |
|  | Chemokine (C-C motif) ligand 5 | CCL5 | F: CCTGCTGTTTTGCCTACCTC  R: GCACTTGCTGCTGGTGTAGA |  |
|  | Chemokine (C-C motif) ligand 20  Macrophage inflammatory protein 3 α | CCL20 | F: CTGCAGCAAGTCAGAAGCAG  R: CTGTGTGAAGCCCACAAGAA | F: AGCCTGTGACATCAATGCAG  R: GCTTGCTTCACCCACTTCTT |
|  | Interleukin 1 receptor antagonist | IL1RN | F: GAAAGAGACCCTGCGAGATG  R: CTTCTTGCAAGTATCCAGCAAC | F: TGTGGTACCCATCGAACCTC  R: TTGAGCTTGATCTCATCTCCAG |

| **Gene group** | **Gene name** | **Abbreviation** | **Primer sequence 5’ to 3’ (assay 1)** | **Primer sequence 5’ to 3’ (assay 2)** |
| --- | --- | --- | --- | --- |
| **Type I interferon signaling** | Interferon beta 1 | IFNB1 | F: TGCCAGAACCTCCTGTG  R: TCGGTCGTGTCTCCCAT |  |
|  | Signal transducer and activator of transcription 1 | STAT1 | F: CACGAAGGTGATGAACATGG  R: TCTTTCAGTTGCAGGTGTCG | F: CTCAATGTGGACCAGCTGAA  R: TTGTCCATGGAATCAGACCA |
| **Interferon stimulated genes** | Interferon-inducible protein 10 (IP10) | CXCL10 | F: ACGCTGTACCTGCATCGAG  R: GCAGGATTGACTTGCAGGA | F: TGATTCCTGCAAGTCAATCCT  R: GACATCTTTTCTCCCCATTCC |
|  | ISG15 ubiquitin-like modifier | ISG15 | F: CAGTTCATCGCCCAGAAGAT  R: ACTTCCCTGCTGTCAAGGTG | F:ACATCCTGGTGAGGAACGAC  R:CTTGAGCACAGCCACAGTCT |
|  | Interferon stimulated exonuclease 20kDa | ISG20 | F: CAGGTGCTGAAGGGCAAG  R: TGTCGTAGATGGCGTAGCTG | F:CACAGCTCGGTGGAGGAC  R:CACTGCTCTCGAATTCTCCTG |
|  | Interferon-induced protein with tetratricopeptide repeats 1 | IFIT1 | F: GAAGCTCTTCGGCTCTCAAA  R: CTGTCTGGTGATGCTGGAAA | F:ACATTTCAAACGTGCTGTGC  R:CGCTTCTGCGTACATTTCTG |
|  | 2'-5'-oligoadenylate synthetase 1 | OAS1 | F: TATGCCTGGGAACAAGGAAG  R: GATGCAGAGGTCCTGATGCT | F:GCATCAGGACCTCTGCATCT  R:GGGTTTTGCAAGTTGTCTCC |
|  | Interferon, alpha-inducible protein 6 | IFI6 | F: GGTATCGCTGTTCCTGTGCT  R: TGAGCGTCTTTTTCCGTCTT |  |
|  | MX dynamin-like GTPase 1 | MX1 | F: TTTTTCAACCTCCACCGAAC  R: CGGATGGATTTCTCAGCTTC | F:AACTCGTGGGGCAGTATGAC  R:CAACCACAGCACTCCATTTG |
| **Acute phase proteins** | Serum Amyloid A3 | SAA3 | F: CCCTCTGCTCAAGGGTATGA  R: AGCAGGTCTGAAGTGGTTGG | F:AGACTATTCAGGGAATCACAGACC  R:CTTCGAATCCTTCCGTACCTC |
|  | Lactoferrin | LF | F: GGGAGAACACGAATGGAGAG  R: AGGCAGAGCAACCTGAAGTC | F:CGACACAGTCTGGGAGAACA  R:CAGAGCAGCCTGAAGTCCTC |
|  | Haptoglobin | HP | F: TGGATCGTGTGGGTTATGTG  R: CACAGGCAGCATGACGTACT |  |
|  | Lipocalin 2 | LCN2 | F: GAGCTGACCCCTGAAGTGAG  R: TGATGTGGTCATCGGTGAGT | F:GGCAGGGAATGCAATTAAGA  R:TGGAGGTGACGTTGTAGCTG |
| **Apoptosis** | Bcl-2-associated X protein | BAX | F: GCTGGACATTGGACTTCCTT  R: TGGGTGTCCCAAAGTAGGAG | F: GGGTTGTCGCCCTTTTCTAC  R: TGATGGTCCTGATCAACTCG |
|  | B-cell lymphoma 2 | BCL2 | F: ATGTGTGTGGAGAGCGTCAA  R: CGGTTCAGGTACTCGGTCAT | F: CTACGAGTGGGATGCCAGAG  R: GGCTGGGAGGACAGGATG |
|  | Superoxide dismutase 2 | SOD2 | F: GGTTGGCTTGGCTTCAATAA  R: AGCAGGGGGATAAGACCTGT | F: AAGGGTGATGTCACAGCTCA  R: GGGCTCAGATTTGTCCAGAA |
|  | Caspase 3 | CASP3 | F: TCGAGCTCATGCACATTCTT  R: TTGCATGAAAAGCAGAATCG | F: TGGCCTGTCAGAAAATACCA  R: TCGCCAGGAAAAGTAACCAG |

| **Gene group** | **Gene name** | **Abbreviation** | **Primer sequence 5’ to 3’ (assay 1)** | **Primer sequence 5’ to 3’ (assay 2)** |
| --- | --- | --- | --- | --- |
| **Enzymes** | Matrix metallopeptidase 8 | MMP8 | F: GTCTGGAGGAGACACCCTCA  R: AAGCAGCCTGTATGCCATTT | F: CACGCTCCGTGGAGAAATAC  R: CCATTGGTAGCTGAGGGTGT |
|  | Calgranulin B, S100 calcium binding protein A9 | S100A9 | F: CTCAAACAGCTGGTGCAAAA  R: TCCAGGTCCTCCATGATCTC | F: GAGATCATGGAGGACCTGGA  R: GGCCACCAGCATAATGAACT |
|  | Heat shock protein 70.1 | HSPA1A | F: CAAGATCACCATCACCAACG  R: ACCTCGTCCTCTGCCTTGTA | F: CAAGTGCCAGGAGGTGATTT  R: CAGCTCCTTCCTCTTGTGCT |
|  | Stress-inducible phosphoprotein 1 (Hop) | STIP1 | F: TTGACACAGCCTTGAAGCAC  R: GGCTGCTTGGTTGGTTATGT | F: CCGACCTTCATCAAGGGTTA  R: CAGGTCGAGTGCTTTCTGGT |
|  | Annexin A1 | ANAX1 | F: GGCTAAAGACATCGCCTCAG  R: CCTCAGATCGGTCACCCTTA | F: TAAGGGTGACCGATCTGAGG  R: TCATATAAGGCCCTGGCATC |
| **Cell adhesion** | Integrin, alpha M | ITGAM | F: CTTGAGGCCTCCACCAAATA  R: GCCCAGGTTGTTGAACTGAT | F: AAGAGCCCAATGCTGAACTG  R: TCTGGAGGGTGACCTTGAAC |
|  | Toll like receptor adapter molecule 1 | TICAM1 | F:CTATCCGGTGGAGTGTACGG  R: GACTGGCGTCTGATCTGTGA | F: AACCCGAGGAGATGAGCTG  R: GGTAGTCACCTCGGGAAGC |
|  | Integrin β2 | ITGB2 | F: CATCCTCCTGCTGGTCATCT  R: TCTCGAAGCGATGGTACTCC | F: CATGCTCGAGTGTGTGAAGG  R: CAGATGACCAGCAGGAGGAT |
| **Other immune related genes** | Major histocompatibility complex,  class 2 | MHCII | F: TCGAAATGGCATACCTGTCA  R: AGGAAGGGGAGGTAGTGGAA | F: GCCCACAACAGAGGATGTCT  R: AGGGGCTGGAGCTTCATACT |
|  | Complement C3 | C3 | F: AGGCCACTTTCATGGTGTTC  R: GGATGGACACATCCAGGTTC | F: AGCTGAACCTGGATGTGTCC  R: GCAGATTCCCAGAGGATACG |
| **Reference genes** | 5’-aminolevulinate synthase 1 | ALAS1 | F: CGCCTTTGAAACTGTCCACT  R: ATCCACGAAGGTGATTGCTC |  |
|  | Heat Schock protein 90 (cytosolic) | HSP90AA1 | F: TCGCAGAGTTTTCATCATGG  R: CCTCAGAATCCACCACACCT |  |
|  | Acetyl-CoA carboxylase alpha | ACACA | F: CACCACCCCGTTCAGTATTT  R: CTGTACCCGTCATTGCCTTT |  |
|  | Hydroxymethylbilane synthase | HMBS | F: AGGATGGGCAACTGTACCTG  R: GTGGTTTGCATGGTGTCTTG |  |
|  | Actin, beta | ACTB | F: CTCTTCCAGCCTTCCTTCCT  R: TAAAGGTCCTTGCGGATGTC |  |
|  | Hypoxanthine phosphoribosyltransferase 1 | HPRT1 | F: GCAGACTTTGCTTTCCTTGG  R: ACACTTCGAGGGGTCCTTTT |  |
|  | Glyceraldehyde-3-phosphate dehydrogenase | GAPDH | F: CGACTTCAACAGCGACACTC  R: TGCTGTAGCCGAATTCATTG |  |
